# Swallowing improvement surgeries

**DOI:** 10.1007/s00405-024-08452-z

**Published:** 2024-01-24

**Authors:** Carmel Cotaoco, Rumi Ueha, Misaki Koyama, Taku Sato, Takao Goto, Kenji Kondo

**Affiliations:** 1 Department of Otolaryngology and Head and Neck Surgery, The University of Tokyo, Tokyo, Japan; 2 Ear Nose Throat Head and Neck Surgery Institute, The Medical City, Pasig, Philippines; 3 Swallowing Center, The University of Tokyo Hospital, 7-3-1 Hongo, Bunkyo-Ku, Tokyo, 113-8655 Japan

**Keywords:** Dysphagia, Swallowing, Surgery, Cricopharyngeal myotomy, Laryngeal suspension

## Abstract

**Purpose:**

To discuss the different swallowing improvement surgeries that address one or more dysfunctional pharyngolaryngeal structures causing dysphagia. These surgeries reduce the risk of aspiration without sacrificing vocal function.

**Methods:**

We searched the PubMed database and used Google Scholar search engine to find studies discussing the different swallowing improvement surgeries. A manual search of references in selected articles and reviews was done as well. No chronologic limitation was set for the studies; however, only articles written in English and Japanese were considered. Due to the nature of this article, no particular inclusion or exclusion criteria were set when searching for studies to be used as references; however, all relevant studies were reviewed and agreed upon by the authors for inclusion in this review article.

**Results/discussion:**

Surgeries to improve swallowing function can be categorized into those that reinforce nasopharyngeal closure or pharyngeal contraction, improve laryngeal elevation or pharyngoesophageal segment opening, and those that improve vocal fold closure to protect the airway during swallowing. They are an effective alternative treatment that may significantly improve these patients’ quality of life. Swallowing rehabilitation with the altered pharyngolaryngeal structures is required post-operatively to significantly improve patients’ dysphagia.

**Conclusions:**

Surgeries to improve swallowing function address specific dysfunctional sites involved in the swallowing mechanism. Choosing the most appropriate surgery for each patient requires knowledge of the pathophysiology for their dysphagia and detailed pre-operative work-up.

## Introduction

The swallowing mechanism is complex and requires the coordinated movement of many structures. Dysphagia occurs when one or more of these structures is not functioning properly. If not addressed, it may lead to serious sequelae, such as malnutrition, aspiration pneumonia, or even death. The goal of all treatments for dysphagia is to ensure a safe swallow and improve patients’ quality of life [1].

**Fig. 1:**
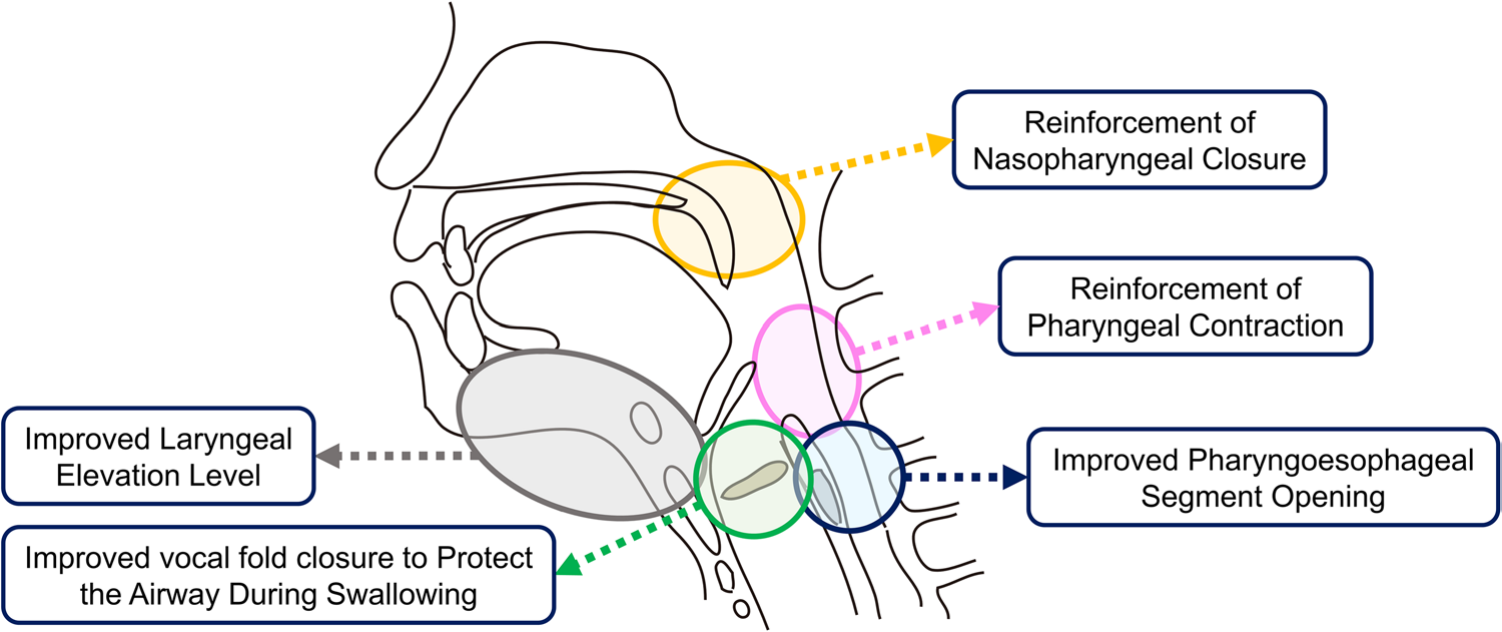
Anatomic sites addressed with swallowing improvement surgeries. Swallowing improvement surgeries focus on changing laryngeal, pharyngeal, and upper esophageal structures, depending on the underlying cause of dysphagia

The first line of treatment for dysphagia is swallowing therapy and diet modification; however, this may not be effective for all patients. In these cases, surgery to improve swallowing function should be considered to address deficient sites of the swallowing mechanism or to supplement non-surgical interventions [1, 2]. These focus on changing laryngeal, pharyngeal, and upper esophageal structures, depending on the underlying cause of dysphagia (Fig. 1, Table 1). Others have proposed a similar classification of these surgeries according to anatomical location [2]; however, this paper will present these surgeries from a more functional classification. Specifically, these surgeries have been classified to address the following:

- Reinforcement of nasopharyngeal closure.
- Reinforcement of pharyngeal contraction.
- Improved laryngeal elevation level.
- Improved pharyngoesophageal segment opening.
- Improved vocal fold closure to protect the airway during swallowing.

**Table 1:** Summary of surgeries to improve swallowing function

Purpose	Types of surgeries	Description
(A) Reinforcement of nasopharyngeal closure	Palatopexy [2, 3]	Approximation of soft palate to posterior nasopharyngeal wall with decreased diameter of velopharyngeal opening
	Push-back surgery [4]	Mobilizing mucoperiosteal flaps of the palate to lengthen the soft palate with subsequent decrease in the size of the velopharyngeal opening
	Pharyngeal flap [5–7]	Insertion of a pharyngeal flap into the soft palate to close the defective part of the velopharynx. The flap may be superiorly, inferiorly, or laterally based
(B) Reinforcement of pharyngeal contraction	Hypopharyngeal pharyngoplasty	Resection of pyriform sinus mucosa with advancement and suturing of the inferior constrictor muscles to thyroid cartilage [12]
		Pharyngeal suture with resection of the pharyngeal wall [9]
		Submucosal suturing of the pyriform sinus without pharyngeal wall incision and pharyngeal wall reinforcement with polypropylene mesh [10]
		Lateral 1/3 of the thyroid ala on the affected side is resected and the pyriform sinus is sutured (lateral thyrolaminectomy) [11]
(C) Improved laryngeal elevation level	Hyo-mandibular suspension [14, 15]	Hyoid bone is approximated to the mandible, restoring continuity of hyomandibular complex
	Laryngeal suspension	
	Thyro-hyoido-pexy [11, 16]	Thyroid cartilage is approximated to the hyoid bone, pushing the epiglottis back into a retroflexed position and shifting the larynx antero-superiorly
	Thyro-mandibular suspension [17]	Thyroid cartilage is approximated to the mandible, shifting the larynx antero-superiorly
	Thyro-hyoido-mandibular suspension [16, 18]	Thyroid cartilage is approximated to the hyoid bone and the hyoid bone is approximated to the mandible, shifting the larynx antero-superiorly
(D) Improved pharyngoesophageal segment opening	Pharyngoesophageal dilation [20−21]	Circumferential splitting and stretching of stenotic areas at the pharyngoesophageal area using mercury-filled bougies, wire-guided polyvinyl dilators, or balloon dilators under endoscopic or fluoroscopic guidance
	Cricopharyngeal myotomy [26−27]	Incision through all fibers of the cricopharyngeus muscle, including some fibers of the inferior constrictor and cervical esophagus muscles, through an external approach
	Endoscopic cricopharyngeal myotomy [30−31]	Incision through all fibers of the cricopharyngeus muscle through a transoral, endoscopic approach, most often done with a CO2 laser
	Zenker’s diverticulum surgery [34, 35]	External approach: Excision or suspension of the diverticular sac
		Endoscopic approach: The common wall between the diverticulum and esophagus is divided using electrocautery, CO2 laser, or a stapling device
(E) Improved vocal fold closure to protect the airway during swallowing	Vocal fold injection [36−37]	Injection of implant material (collagen, autologous fat, hyaluronan, calcium hydroxylapatite, or fibroblastic growth factor) into the paraglottic space to medialize the vocal fold
	Laryngeal framework surgery	
	Type I thyroplasty [44−45]	A laryngoplasty window is created on the thyroid cartilage at the level of the vocal folds, and an implant is then placed to medialize the vocal fold
	Arytenoid adduction [48−49]	The muscular process of the arytenoid is sutured and secured anteriorly to the apex of the thyroid cartilage, simulating the action of the lateral cricoarytenoid muscle

The purpose of this paper is to review the different surgeries to improve swallowing function and disseminate more detailed information about them to medical professionals.

## Methods and ethics

We searched the PubMed database and used the Google Scholar search engine to find studies discussing the different swallowing improvement surgeries. A manual search of references in selected articles and reviews was done as well. Due to the nature of this article, no particular inclusion or exclusion criteria were set when searching for studies to be used as references. No chronologic limitation was set for the studies; however, only articles written in English and Japanese were used for this review. All relevant studies were reviewed and agreed upon by the authors for inclusion in this review article. Although ethical issues are not applicable to narrative reviews, this study protocol was approved by the Human Ethics Committee of the University of Tokyo Hospital (No. 2022179NI) and complied with the tenets of the amended Declaration of Helsinki. Specifically, written informed consent was obtained from every patient whose treatment was presented as an illustrative case in the last section of this paper, and patient anonymity was preserved.

## Results/discussion

### Pathophysiology

The disease background of patients with dysphagia is varied, and treatment options must be selected accordingly. Various surgical treatments have been applied for different pathological conditions (Fig. 1). In addition to a detailed physical examination and airway assessment, a detailed site-specific evaluation of swallowing status with swallowing function tests (fiberoptic endoscopic evaluation of swallowing, videofluoroscopic swallowing study, and high-resolution manometry) is mandatory to develop a treatment plan.

### Surgical management for dysphagia

In the following sections, surgeries to improve swallowing function while preserving speech function in the adult population will be described and explained briefly (Table 1).

### Types of surgeries based on functional purpose

**Fig. 2:**
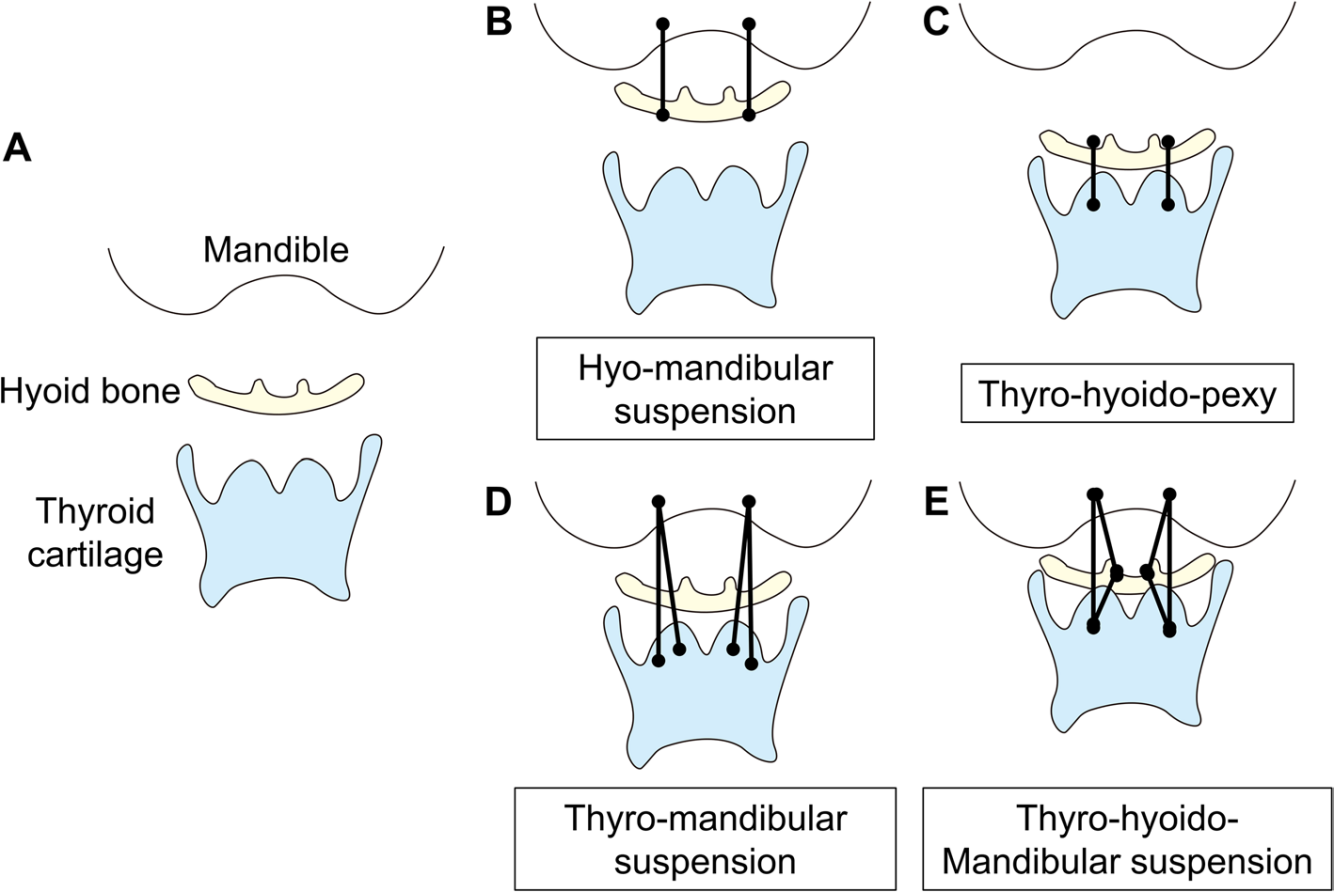
Surgeries to improve laryngeal elevation. Normal anatomy (**a**). Approximation of hyoid to mandible (**b**), thyroid cartilage to hyoid (**c**), thyroid cartilage to mandible (**d**), and thyroid cartilage to hyoid and hyoid to mandible (**e**)

1. Reinforcement of nasopharyngeal closure
  Palatopexy, push-back surgery, and pharyngeal flap are surgeries to reinforce nasopharyngeal closure. Palatopexy involves suturing the soft palate to the posterior nasopharyngeal wall [2, 3], while push-back surgery utilizes mucoperiosteal flaps of the palate to lengthen the soft palate [4]. In this section, we focus on pharyngeal flap surgery as a treatment for dysphagia.
  - Pharyngeal flap
    Overview
      In 1973, Hirano et al. first reported performing cricopharyngeal myotomy with a pharyngeal flap for dysphagia secondary to Wallenberg syndrome [5]. The most common approach is the use of a centrally located pharyngeal flap with lateral ports for air to pass, but a lateral pharyngeal flap may be more appropriate for patients with a lateral velopharyngeal gap [6, 7].
      In the lateral pharyngeal flap, the soft palate is incised in the midline, and its undermined posterior mucosa is sutured to a laterally based pharyngeal flap created only on the affected (paralyzed) side. The soft palate is then closed primarily, and the defect on the posterior pharyngeal wall is covered with a polyglycolic acid sheet and fibrin glue [6].
    Expected results
      Laterally based pharyngeal flaps are designed to close the defective part of the velopharynx [7] and prevent the reflux of food into the nasal cavity. The centrally located flaps may impair the motion of the unaffected side of the pharynx and allow for the escape of pharyngeal pressure through the affected side during swallowing [6].
    Complications
      Complications are rare but may include flap dehiscence, nasal obstruction, snoring, hyponasality, obstructive sleep apnea, or airway obstruction [4].
2. Reinforcement of pharyngeal contraction
  - Hypopharyngeal pharyngoplasty
    Overview
      In 1948, Naffziger first described suturing the fascia of the carotid sheath to the midline of the larynx and cricoid to increase the stiffness of the pharyngeal wall with suturing of the posterior belly of the digastric muscle to the thyroid cartilage [8]. This was the first report of such surgery to improve swallowing function. Since then, many variations have been described.
      First, it is possible to perform a full-thickness resection of a portion of the pharyngeal wall with suturing of the defect on the paralyzed side [9]. This may be additionally reinforced with a polypropylene mesh [10]. Alternatively, resection of the lateral 1/3 of the thyroid cartilage with suturing of the pyriform sinus (lateral thyrolaminectomy) may be done [11]. Another approach involves resection of pyriform sinus mucosa with advancement and suturing of the inferior constrictor muscles to the thyroid cartilage anteriorly [12].
    Expected results
      The use of hypopharyngeal pharyngoplasty is most commonly reported in patients with unilateral paralysis of the pharyngeal wall, seen in conditions, such as Wallenberg syndrome or unilateral cranial nerve injuries. It is expected to improve the tone or tension of the paralyzed pharyngeal wall and decrease dead space for secretions and food residue to collect by reducing pyriform sinus mucosa. These changes improve the propulsion of the food bolus into the esophagus [2, 13].
    Complications
      Although complications with this procedure are rare, possible complications include an anastomotic leak and infection [12].
3. Improved laryngeal elevation level (Fig. 2)
  - Hyo-mandibular suspension
    In 1959, both Edgerton and McKee and Desperez and Kiehn reported on the use of a suspensory tie and fascia lata strips, respectively, connecting the hyoid bone to the mandible for cases of extensive head and neck malignancies that disrupted the hyomandibular complex. Hyo-mandibular suspension is useful in cases where the suprahyoid muscles or hypoglossal nerve is disrupted. It approximates the hyoid bone to the mandible and restores the integrity of the hyomandibular complex [14, 15].
  - Laryngeal elevation surgery (Thyro-hyoido-pexy, thyro-mandibular suspension, thyro-hyoido-mandibular suspension)
    Overview
      Laryngeal elevation surgery (laryngeal suspension) was first described in 1948 by Naffziger who sutured the posterior belly of the digastric muscle to the thyroid cartilage in a patient with unilateral paralysis of the ninth-to-twelfth cranial nerves [8]. Variations of laryngeal suspension were subsequently developed, which involved the suspension of either the hyoid bone [11, 16], thyroid cartilage [17], or both [16, 18] to the mandible (Fig. 2). Suspension is done using a variety of materials, including non-absorbent and absorbent threads, steel wires, polytetrafluoroethylene (Teflon) tape, or expanded polytetrafluoroethylene (Goretex).
      A thyro-hyoido-pexy compresses the tissues in the preepiglottic space and pushes the epiglottis into a retroflexed position, offering protection of the glottic opening during swallowing [11, 16]. Thyro-mandibular suspension elevates the larynx without hindering the mobility of the hyoid bone [17]. Thyro-hyoido-mandibular suspension is used in cases with severely impaired laryngeal elevation, where the thyroid cartilage is approximated to the hyoid bone and the hyoid bone is then approximated to the mandible [16, 18].
      Transection of the infrahyoid muscles is often done in conjunction with this surgery to prevent downward traction of the larynx [16], as well as cricopharyngeal myotomy to further improve the passage of the food bolus [18, 19].
    Expected results
      The purpose of laryngeal elevation is to lift the laryngeal framework upward and anteriorly, approximating it more closely to the hyoid bone or mandible. This improves the closure of the laryngeal vestibule by laying the epiglottis backward, and it contributes to the opening of the upper esophageal sphincter (UES), assisting the passage of the food bolus into the esophagus [18].
    Complications
      Complications include damage or infection of the thyroid cartilage and airway obstruction by the epiglottis and base of the tongue if the larynx is lifted excessively or due to laryngeal edema; therefore, concurrent tracheostomy is recommended [17, 18].
4. Improved pharyngoesophageal segment opening
  - Pharyngoesophageal dilation
    Overview
      Pharyngoesophageal dilation became more widespread in 1902, when Chevalier Jackson designed a distally lighted esophagoscope which allowed relatively safer antegrade bougienage through the oral cavity under direct visualization [20]. Later on, retrograde [21] (via gastrostomy tract) and combined antegrade and retrograde approaches were developed and done under endoscopic or fluoroscopic guidance to improve the safety and success of this procedure [22].
      Pharyngoesophageal dilation is most commonly done for strictures in the pharyngeal, cricopharyngeal, or upper cervical esophageal areas. In all approaches, the stenotic segment is identified and a dilator is passed through the lumen in gradually increasing diameters to slowly dilate it [22]. Dilators used include mercury-filled bougies, wire-guided polyvinyl dilators, or controlled radial expansion balloon dilators, which are usually left in place for a few minutes. In general, the rule is not to pass more than 3 dilators of sequential size once moderate resistance is felt [23]. Others consider the identification of a mucosal tear during the procedure as a sign to cease further dilation [24].
    Expected results
      Pharyngoesophageal dilation essentially splits stenotic segments in the pharyngeal or upper esophageal areas and causes circumferential stretching, facilitating the passage of the food bolus through this area. It should be noted that the effects of the dilation are most often temporary and need to be performed as a series of dilations [22].
    Complications
      Possible complications include pharyngeal or esophageal tears or perforations, which may lead to mediastinitis, pneumothorax, pneumomediastinum, or pharyngocutaneous fistula formation [25].
  - Cricopharyngeal myotomy
    Overview
      Kaplan in 1951 is generally considered to be the first to have performed a cricopharyngeal (CP) myotomy through an external approach for patients with hypopharyngeal paresis due to bulbar poliomyelitis. Since then, this technique has been applied widely to address oropharyngeal dysphagia associated with neurogenic, myogenic, structural, and idiopathic diseases [26, 27].
      In the external approach, the CP muscle is exposed and may be stretched in a variety of ways (esophagoscope, bougies, balloons, etc.) to better expose its fibers, which are then transected up to the mucosa. Many surgeons advocate to extend the myotomy 1–2 cm cranially and caudally to include muscle fibers from the inferior constrictor and cervical esophagus, as these contribute to the UES as a unit [27–29].
    Expected results
      CP myotomy assists in the passage of the food bolus by decreasing the resistance of the UES and increasing the length of time that food may pass through the sphincter, instead of being limited to the brief relaxation time in a normal swallow [28, 29].
    Complications
      Complications that may occur with an external approach are esophageal perforation, recurrent laryngeal nerve injury, pharyngocutaneous fistula formation, or wound infection [26]. Many also consider severe gastroesophageal reflux a contraindication to CP myotomy, as the protective function of the UES is lost and refluxate material from the esophagus may spill into the hypopharynx [18, 28].
  - Endoscopic cricopharyngeal myotomy
    Overview
      An endoscopic or transmucosal approach to CP myotomy using electrocautery was done by Dohlman and Mattsson as early as 1960 as part of the treatment for Zenker’s diverticulum [30]. In 1994, Halvorson and Kuhn performed an endoscopic CP myotomy using a potassium-titanyl-phosphate (KTP) laser [31]. Since then, the carbon dioxide (CO2) laser has increased in popularity and is more frequently used to incise the CP muscle [32].
      Once the CP muscle is exposed and identifiable as a posterior bulge, often called “the cricopharyngeal bar”, it is cut transmucosally in the midline layer by layer until the buccopharyngeal fascia [32] or prevertebral fatty tissue [30] is seen.
    Expected results
      The expected results are similar to that of the external approach; however, it avoids the scar seen with an external approach and allows for a shorter operating time, quicker recovery, and shorter hospital stay [32].
    Complications
      The most common complication associated with an endoscopic approach is an esophageal perforation with entry into the neck spaces and possible abscess formation or mediastinitis [26, 30]. It should also be noted that this approach is not suitable for patients that have difficulty with neck extension, as it would be difficult to expose and visualize the cricopharyngeal area transorally [33].
  - Zenker’s diverticulum surgery
    Overview
      Zenker’s Diverticulum (ZD) may be approached externally through simple excision (diverticulotomy) or suspension (diverticulopexy) of the pouch. In the endoscopic approach, the common wall between the diverticulum and esophagus is exposed transorally and divided using electrocautery, a carbon dioxide laser, or a stapling device [34]. Although the endoscopic technique is said to be simpler with a shorter operating time, its use is precluded in cases where the diverticulum cannot be visualized adequately [35].
    Expected results
      In all approaches to surgery for ZD, the goal is to prevent the accumulation of food and other debris in the pouch. Concurrent cricopharyngeal myotomy is said to decrease the recurrence of the diverticulum, as the pressure at the UES is decreased [35].
    Complications
      Possible complications include pharyngocutaneous fistula or abscess formation, esophageal perforation, mediastinitis, or pneumomediastinum. Recurrent laryngeal nerve injury is of particular risk with diverticulostomy [35].
5. Improved vocal fold closure to protect the airway during swallowing (medialization of the vocal fold).
  - Vocal fold injection
    Overview
      Injection laryngoplasty (IL) may be done using a variety of materials, including bovine [36] and human [37] collagen, autologous fat [38], hyaluronan [39], calcium hydroxylapatite [40], and fibroblastic growth factor (FGF) [41]. To this day there is no ideal injection material, and the problem of resorption remains a challenge.
      In patients with unilateral vocal fold paralysis that experience dysphagia, the injection is applied to the paraglottic space to medialize the entire vocal fold. The adequate amount of injected material can be confirmed visually and by asking the patient to phonate (for awake procedures) [42, 43].
    Expected results
      IL is used in cases of unilateral vocal fold paralysis with glottic insufficiency, resulting in improved glottic closure and decreasing the chances of aspiration during swallowing. However, some postulate that improved glottic closure may also increase the intrapharyngeal pressure during swallowing, triggering the swallow reflex and improving bolus clearance [42].
    Complications
      The most common complications from IL are extrusion, migration, inflammation, and granuloma formation due to the injected substances [42].
  - Laryngeal framework surgery
    Overview
      **Medialization (type I) thyroplasty** became the preferred treatment for unilateral vocal fold paralysis when Isshiki reported on it in 1974 [44]. Medialization thyroplasty is done by creating a window on the thyroid ala of the affected side at the level of the vocal folds to access the paraglottic space. Once the window is created, an implant [silicone [45], expanded polytetrafluoroethylene (Gore-Tex) [46], fascia [47], etc.] is placed into the paraglottic space to medialize the entire vocal fold. Correct positioning can be confirmed by asking the patient to phonate and visually using a flexible videolaryngoscope [48].
      **Arytenoid adduction** is usually done concurrently with medialization thyroplasty. The muscular process of the arytenoid is identified, and a suture is passed through it then secured anteriorly to the apex of the thyroid cartilage. This essentially simulates the action of the lateral cricoarytenoid muscle [48, 49].
    Expected results
      Expected results are the same as for IL, but arytenoid adduction is more effective in cases with a vocal fold height difference and a wide posterior glottic gap [1, 48].
      The effect of a Type I thyroplasty is permanent although reversible since the implant material is not resorbed [48].
    Complications
      Complications in Type I thyroplasty include extrusion, migration, or foreign body reaction to the implant, as well as airway compromise if there is impaired motion of the contralateral vocal fold. Arytenoid adduction has a higher risk for airway compromise due to edema of the posterior glottis, and pyriform sinus perforation is another associated but rare complication [48].

It must be emphasized that improved swallowing function cannot be achieved immediately through surgery alone. Rehabilitation of the modified pharyngolaryngeal complex with swallowing exercises in the post-operative period is necessary.

### Choosing the appropriate surgery for patients with severe dysphagia

Here, we provide some specific examples to illustrate in which clinical situations it is appropriate to use the different swallowing improvement surgeries described in the article.

**Fig. 3:**
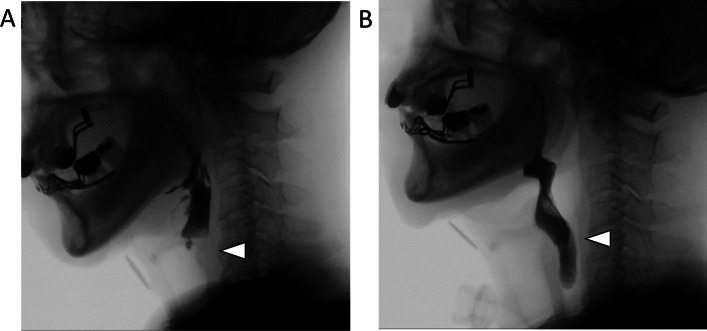
Videofluoroscopic swallowing study findings. **a** Pre-operative: inability of contrast to flow past upper esophageal sphincter (UES, white arrowhead); **b** post-operative: contrast flows smoothly past UES (white arrowhead)

**Fig. 4:**
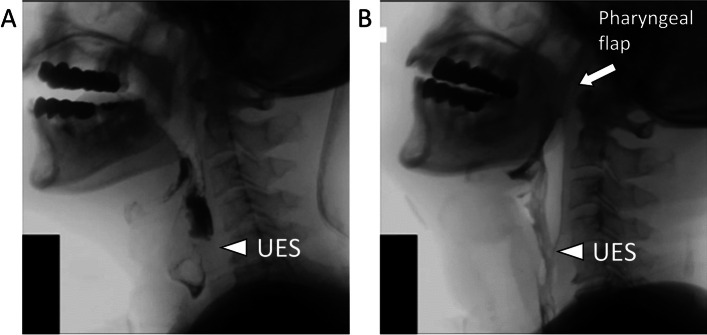
Videofluoroscopic swallowing study findings. **a** Pre-operative: inability of contrast to flow past upper esophageal sphincter (UES, white arrowhead); **b** post-operative: pharyngeal flap (white arrow) with contrast flowing past UES (white arrowhead)

**Fig. 5:**
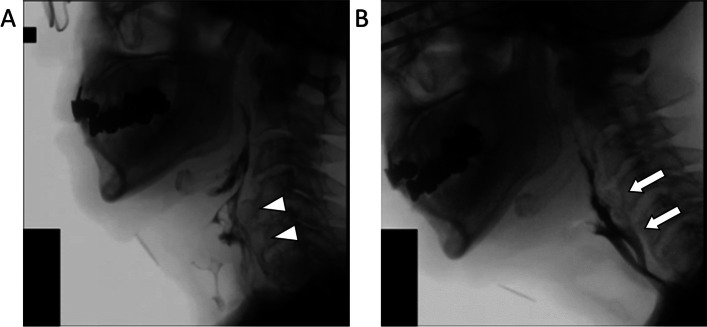
Videofluoroscopic swallowing study findings. **a** Pre-operative: excessive osteophyte (white arrowheads) at cervical spine; **b** post-operative: osteophyte reduced significantly (white arrows) with improved flow of contrast to esophagus

1. Dysphagia in a 66-year-old man with traumatic head injury leading to a subarachnoid hemorrhage and skull base fracture with multiple lower cranial nerve deficits. Swallowing evaluation revealed severely impaired pharyngeal contraction and laryngeal elevation, as well as impaired bolus passage through the UES. Paralysis (paramedian position) and atrophy of the right vocal fold was also seen with insufficient glottic closure. Nasopharyngeal valve closure was only slightly impaired. A bilateral cricopharyngeal myotomy, thyro-mandibular suspension, and autologous fat injection at the right vocal fold was performed for this patient, and his oral intake status improved post-operatively (Fig. 3).
2. Dysphagia in a 62-year-old man with Wallenberg syndrome. The patient had severe left-sided velopharyngeal insufficiency, left vocal fold paralysis (median position), poor laryngeal elevation, severely decreased pharyngeal contraction, and impaired bolus passage through the UES. Patient's oral intake status improved after a lateral pharyngeal flap on the left, thyro-mandibular suspension, and bilateral cricopharyngeal myotomy (Fig. 4).
3. Dysphagia in a 76-year-old man after contracting COVID-19. One year after COVID-19 treatment, he still had impaired laryngeal elevation, decreased pharyngeal contraction, an excessive osteophyte at the cervical spine, and impaired bolus passage through the UES. His oral intake status improved after thyro-mandibular suspension, removal of cervical spine osteophyte, and left-sided cricopharyngeal myotomy (Fig. 5).

## Conclusion

Surgeries to improve swallowing function address specific dysfunctional sites involved in the swallowing mechanism, namely the velopharyngeal closure, pharyngeal wall contraction, pharyngoesophageal inlet opening, laryngeal elevation level, or vocal fold closure. Choosing the most appropriate surgery for each patient requires knowledge of the pathophysiology for their dysphagia and detailed pre-operative work-up. Swallowing rehabilitation with the altered pharyngolaryngeal structures is required post-operatively to significantly improve patients’ dysphagia.

## Data Availability

The datasets used in this paper are available from the corresponding author upon reasonable request.
